# Early Morbidities of Hypoxia-Ischemic Encephalopathy in Term Neonates With a Resistive Index as a Prognostic Indicator

**DOI:** 10.7759/cureus.61936

**Published:** 2024-06-08

**Authors:** Gowthami G S, Ravi Kumar Yeli, Vishal Nimbal, Dhanya S B, Praveen Kumar M

**Affiliations:** 1 Department of Pediatrics, Al-Ameen Medical College and Research Center, Vijayapura, IND; 2 Department of Radiology, Bijapur Lingayat District Educational (BLDE) (Deemed to Be University), Shri B M Patil Medical College Hospital and Research Centre, Vijayapura, IND; 3 Department of Radiodiagnosis, Bijapur Lingayat District Educational (BLDE) (Deemed to Be University), Shri B M Patil Medical College Hospital and Research Centre, Vijayapura, IND; 4 Department of Radiology, Jagadguru Sri Shivarathreeshwara (JSS) Medical College, Mysore, IND; 5 Department of Radiology, Sri Jayadeva Institute of Cardiovascular Sciences and Research, Mysore, IND

**Keywords:** ultrasound, hie, prognosis, neurodevelopment, ri, cerebral circulation

## Abstract

Background

Acidosis, hypoxemia, and hypercarbia are symptoms of a syndrome known as perinatal asphyxia that occurs during the first and second stages of labor and shortly after delivery due to poor gas exchange. The Doppler technique is a non-invasive way to assess the risk of neurodevelopment damage in hypoxic-ischemic encephalopathy (HIE) that may be done at the patient's bedside without disturbing them. The study aims to evaluate cranial ultrasound findings in HIE and investigate the role of resistive index (RI) values assessed by color Doppler transcranial ultrasonography in predicting early morbidities in neonates with HIE within 72 hours of life.

Methodology

Prospective observational research was carried out at the north Karnataka region's tertiary newborn critical care unit. The study included 54 infants with HIE in total. The male-to-female ratio was 1.7:1, with 34 (63%) male and 20 (37%) female newborns.

Results

About 32 instances had grade I HIE, 8 had grade II HIE, and 14 had grade III HIE. In 35 instances (64.81%), the RI was normal; in 19 cases (35.19%), it was abnormal. Increased periventricular density and cerebral parenchyma echo density were common Doppler ultrasonography findings. Roughly 93% of people survived, and 7% of people died from HIE. Seizures (12.96%) and acute renal damage (33.33%) were the most frequent consequences.

Conclusion

In instances of HIE, the RI was revealed to be a favorable predictive indicator for newborn prognosis. Counseling and educating parents about early morbidities, anticipated long-term consequences, and the need for follow-up will all benefit from it. Additionally, color Doppler is a practical and secure diagnostic method for determining a newborn's level of HIE.

## Introduction

Severe birth asphyxia, as defined by the World Health Organization (WHO) in the International Classification of Disease, Tenth Revision (ICDS10), is characterized by an APGAR score of 0-3 at one minute of life. An APGAR score of 4-7 at one minute indicates mild to moderate birth asphyxia. Approximately 25% of newborns with hypoxic-ischemic encephalopathy (HIE) suffer long-term neurological impairments, and 15-20% of them do not survive [1,2]. The APGAR score, cord blood pH (pH <7.20 in the umbilical artery), and neonatal acidosis (pH <7.3 in capillary blood) within the first hour of life are limited in predicting long-term outcomes [3-6]. The application of more sensitive techniques, such as neuroimaging, is constrained by expertise and cost [7]. Despite significant advancements in neonatal intensive care reducing the number of children with poor neurodevelopmental outcomes, birth asphyxia remains the leading cause of hypoxic-ischemic brain damage in term neonates [8].

The measurement of the resistive index (RI) and end-diastolic flow velocity (EDFV) in the anterior cerebral artery, as per the American Academy of Neurology and the Child Neurology Society guidelines, is essential for evaluating cerebral perfusion and predicting early outcomes [9]. Hypoxia and increased pCO_2_ levels result in vasodilation, indicated by an increase in EDFV [10,11]. Poor cerebral circulation is identified as the primary cause of hypoxic-ischemic brain injury in newborns. Studies in high-income countries show that a reduced cerebral RI can distinguish asphyxiated neonates from healthy controls and predict neurodevelopmental harm. The RI is measured within the first 72 hours of life using pulse wave Doppler ultrasonography on the sagittal plane anterior cerebral artery, calculated as RI = (S - D)/S, where S is the peak systolic velocity and D is the end-diastolic velocity. A normal RI range is 0.56 to 0.80 [12].

In HIE, the loss of cerebral autoregulation may lead to decreased or absent diastolic cerebral artery flow, resulting in either an elevated RI (>0.80) or a reduced RI (<0.56) due to arterial vasodilation-induced diastolic flow. This study aims to evaluate the predictive validity of RI values using color Doppler transcranial ultrasonography within 72 hours of birth in newborns with HIE, assessing early morbidities and cranial ultrasonography results. The findings aim to enhance diagnosis and treatment strategies, ultimately improving outcomes and care for affected infants.

## Materials and methods

Study design and setting

This prospective observational study was conducted at the tertiary neonatal critical care unit of Bijapur Liberal District Education Association (BLDEA) (Deemed to Be University), Shri B M Patil Medical College Hospital and Research Centre, Vijayapura in the north Karnataka region. The research commenced following ethical clearance from the Institutional Ethical Committee of Shri B M Patil Medical College Hospital and Research Centre, Vijayapura.

Selection Criteria

The study included term neonates (gestation length of at least 37 weeks) who either did not cry at birth or had an APGAR score of 5 or higher at five minutes. These neonates required positive pressure ventilation for at least one minute and were admitted to the neonatal intensive care unit (NICU) within 72 hours of delivery. Excluded from the study were outborn neonates, those with significant congenital malformations, and those with serious illnesses and unstable vital signs. Parents provided signed, informed consent at the time of enrollment. Detailed clinical histories and examination results were recorded for each case. The modified Sarnat and Sarnat [13] grading system was used for HIE staging, categorizing the neonates into three groups based on the severity of HIE namely mild, moderate, and severe.

Data sources and variables

A transcranial Doppler ultrasonography examination was performed on neonates recruited within 72 hours of birth using a Sonosite ultrasound machine. Curvilinear (8-5MHz) and linear (8-4MHz) probes were utilized with neurosonography settings. The right and left anterior cerebral arteries (ACA), located in front of the corpus callosum genu, were assessed to measure the cerebral blood flow characteristics of RI in each patient. Bilateral parasagittal plane imaging of the ACA was performed via the anterior fontanel. An RI value of 0.56 to 0.80 was considered normal, while values below 0.56 or above 0.80 were considered abnormal. Concurrently, other cerebral ultrasonography abnormalities related to HIE were also assessed. A radiologist verified the results of the transcranial Doppler ultrasonography conducted by a senior pediatrician and a neonatology fellow. Early morbidities, such as mortality before discharge, seizures, acute kidney injury, sepsis, shock, and abnormal neurological findings, were evaluated in all the neonates.

Statistical analysis

Statistical analysis was conducted using data entered into a Microsoft Excel sheet (Microsoft Corporation, Redmond, United States), with results presented in counts and percentages. This approach allowed for a thorough examination of the dataset, enabling the identification of trends, patterns, and associations pertinent to the study objectives.

## Results

Table 1 summarizes the distribution of RI values among 54 patients. For the right anterior cerebral artery (ACA), 24.07% (13) of patients had a RI below 0.56, 64.81% (35) fell within the range of 0.56 to 0.80, and 11.11% (6) had a value exceeding 0.80. Similarly, for the left ACA, 27.78% (15) exhibited a RI below 0.56, 64.81% (35) fell within the range of 0.56 to 0.80, and 7.41% (4) had a value exceeding 0.80.

**Table 1:** The resistive index distribution (n=54) ACA: anterior cerebral artery; n: number of patients; %: percentage of patients

Resistive index	Number of patients (n)	Percentage (%)
Right ACA		
<0.56	13	24.07%
0.56 to 0.80	35	64.81%
>0.80	6	11.11%
Left ACA		
<0.56	15	27.78%
0.56 to 0.80	35	64.81%
>0.80	4	7.41%

Table 2 presents the cranial ultrasound findings from the study. Among the patients, 64.81% (35) had normal ultrasound findings, while 22.22% (22) exhibited increased periventricular density. Intraventricular hemorrhage was observed in 12.96% (7) of patients, and increased echodensity of cerebral parenchyma was noted in 25.93% (14) of cases.

**Table 2:** Cranial ultrasound findings n: number of patients; %: percentage of patients

Ultrasound findings	Number of patients (n)	Percentage (%)
Normal	35	64.81%
Increased periventricular density	12	22.22%
Intraventricular hemorrhage	7	12.96%
Increased echodensity of cerebral parenchyma	14	25.93%

Table 3 outlines the immediate neonatal outcomes observed in the study population. Among the patients, 92.59% (50) survived, while 7.41% (4) experienced mortality. Additionally, 55.55% (30) showed improvement, with 33.33% (18) experiencing acute kidney injury, 12.96% (7) developing seizures, and 9.26% (5) presenting with coagulopathy, sepsis, or shock each. Prolonged NICU stay was noted in 16.66% (9) of cases.

**Table 3:** Neonatal immediate outcome ^*^ The observations in patients were not mutually exclusive, as multiple findings could coexist in any given patient. NICU: neonatal intensive care unit; n: number of patients; %: percentage of patients

Neonatal outcome^*^	Number of patients (n)	Percentage (%)
Survivors	50	92.59%
Death	4	7.41%
Improved	30	55.55%
Acute kidney injury	18	33.33%
Seizures	7	12.96%
Coagulopathy	5	9.26%
Sepsis	4	7.41%
Shock	5	9.26%
Prolonged NICU stay	9	16.66%

## Discussion

HIE significantly contributes to early neonatal mortality in asphyxiated preterm or term newborns. The focus on new imaging modalities and biomarkers aims to enhance the early identification, management, and neuroprotection of high-risk infants [14]. HIE results from perinatal asphyxia causing hypoxia and ischemia, which impairs cerebral autoregulation and leads to widespread brain damage, especially in the metabolically active myelinated regions [15].

The study examined 54 neonates diagnosed with HIE, consisting of 34 (63%) males and 20 (37%) females, reflecting a male-to-female ratio of 1.7:1. This distribution aligns with other studies, such as Likitha et al. [16] and Jain et al. [17], which reported ratios of 1.6:1 and 1.5:1, respectively. The severity of HIE was categorized into 32 cases of grade I, 8 cases of grade II, and 14 cases of grade III.

Sonography can sensitively detect hydrocephalus, periventricular leukomalacia, and hemorrhage. The RI of the middle cerebral artery is particularly useful for assessing HIE severity; increased RI indicates impaired autoregulation in severe HIE [15]. In the study, 35 (64.81%) neonates had a normal RI, while 19 (35.19%) had an abnormal RI. In contrast, Kumar et al. [18] found that 50% of neonates with HIE had an abnormal RI, and Jain et al. [17] noted lower RI values in grades II and III HIE, emphasizing the importance of color Doppler USG in assessing cerebral blood flow velocity.

All 19 patients with abnormal RI in the study were associated with grade II and III HIE according to modified Sarnat staging. This is consistent with Barseem et al. [19], who reported that 48.5% of patients had normal cranial ultrasound results, while others had varying degrees of intraventricular hemorrhage, increased periventricular density, and parenchymal echogenicity. The most common complications observed were seizures (12.96%) and acute renal damage (33.33%), aligning with findings by Ahmad et al. [20], where seizures were the most frequent side effect.

Limitations of the study

The study has some limitations, including a relatively small sample size of 54 neonates, which may affect the generalizability of the findings. The male predominance in the sample (63% male to 37% female) might introduce a gender bias, limiting the applicability of the results to the broader population. The study also relies heavily on the RI for assessing the severity of HIE, which, although useful, may not capture all aspects of the condition. The findings regarding RI and its correlation with HIE severity are based on a comparison with other studies, but variations in study designs and methodologies could impact the consistency and comparability of these results.

## Conclusions

In instances of HIE, the RI was revealed to be a favorable predictive indicator for newborn prognosis. Counseling and educating parents about early morbidities, anticipated long-term consequences, and the need for follow-up will all benefit from it. Additionally, color Doppler is a practical and secure diagnostic method for determining a newborn's level of HIE.
